# Telemedicine in Rural India

**DOI:** 10.1371/journal.pmed.0030082

**Published:** 2006-03-07

**Authors:** Sanjit Bagchi

## Abstract

Although nearly 75% of Indians live in rural villages, more than 75% of Indian doctors are based in cities. Might telemedicine be a cheap and fast way to bridge the rural-urban health divide?

In a developing country such as India, there is huge inequality in health-care distribution. Although nearly 75% of Indians live in rural villages, more than 75% of Indian doctors are based in cities [ 1]. Most of the 620 million rural Indians lack access to basic health care facilities [ 2]. The Indian government spends just 0.9% of the country's annual gross domestic product on health, and little of this spending reaches remote rural areas [ 3]. The poor infrastructure of rural health centers makes it impossible to retain doctors in villages, who feel that they become professionally isolated and outdated if stationed in remote areas.

In addition, poor Indian villagers spend most of their out-of-pocket health expenses on travel to the specialty hospitals in the city and for staying in the city along with their escorts [ 4]. A recent study conducted by the Indian Institute of Public Opinion found that 89% of rural Indian patients have to travel about 8 km to access basic medical treatment, and the rest have to travel even farther [ 5].

## Can Telemedicine Bridge the Divide?

Telemedicine may turn out to be the cheapest, as well as the fastest, way to bridge the rural–urban health divide. Taking into account India's huge strides in the field of information and communication technology, telemedicine could help to bring specialized healthcare to the remotest corners of the country [ 6, 7].

The efficacy of telemedicine has already been shown through the network established by the Indian Space Research Organization (ISRO), which has connected 22 super-specialty hospitals with 78 rural and remote hospitals across the country through its geo-stationary satellites. This network has enabled thousands of patients in remote places such as Jammu and Kashmir, Andaman and Nicobar Islands, the Lakshadweep Islands, and tribal areas of the central and northeastern regions of India to gain access to consultations with experts in super-specialty medical institutions [ 8]. ISRO has also provided connectivity for mobile telemedicine units in villages, particularly in the areas of community health and ophthalmology [ 9]. [Fig pmed-0030082-g001]

**Figure pmed-0030082-g001:**
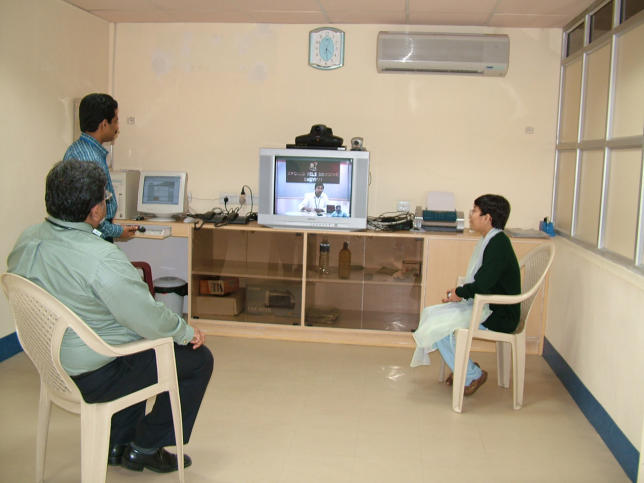
A telemedicine program in action at the Apollo Gleneagles Hospital, Kolkata, India (Photo: Nilmoni Debnath/Apollo Gleneagles Hospital)

This encouraging early success in reaching patients—together with recent technological advances in India, such as the proliferation of fiber optic cables, the expanding bandwidth, and the licensing of private Internet service providers—has encouraged ISRO to set up an exclusive satellite, called HealthSAT, to bring telemedicine to the poor on a larger scale. The proposed satellite would not only serve remote areas of India but also those in other poor countries in Asia and Africa. In the government of India's current budget, INR102.8 billion has been allocated for health [ 10]. HealthSAT is expected to cost only about 1% of this budget, that is, between INR600 million to INR1 billion. Each receiving terminal (where patients and rural doctors are present for audiovisual conferences) in the villages is expected to cost only about INR0.5 million [ 11]. This telemedicine service will save some costs, for example the money that patients would have spent on travel and accommodation.

A telemedicine system in a small health centre consists of a personal computer with customized medical software connected to a few medical diagnostic instruments, such as an ECG or X-ray machine or an X-ray scanner for scanning X-ray photos [ 12]. Through this computer, digitized versions of patients' medical images and diagnostic details (such as X-ray images and blood test reports) are dispatched to specialist doctors through the satellite-based communication link. The information, in turn, is received at the specialist centre where experienced doctors examine the reports, diagnose, interact with the patients (along with local doctors), and suggest appropriate treatment through video-conferencing. The entire system is relatively user-friendly, and only a short period of training is needed for doctors at super-specialty centres and rural health centres to handle the system. And hospital technicians can take care of the operation and maintenance of the equipment.

M. N. Sathyanarayan, Executive Director of Space Industries Development, and organising secretary of the 2005 International Telemedicine Conference, said: “In the pilot phase of the telemedicine project, ISRO is providing telemedicine equipment as well as making available the required bandwidth on INSAT satellites. The main criteria for funding by ISRO are that the hospitals have to be government-run—state or central—or belong to public sector industries. The hospitals have to provide infrastructure as well as doctors and technicians for operating the system.”

“ISRO also provides the equipment and bandwidth to private specialty hospitals and hospitals run by Trusts, if these hospitals provide free service, including specialty consultation to rural hospitals that have been connected in the telemedicine network of ISRO. These hospitals have to provide follow-up treatment to teleconsulted patients at government rates.”

In its telemedicine initiative, ISRO intends to connect different types of Indian health care centers in a series of phases. L. S. Sathyamurthy, Programme Director of Telemedicine at ISRO said: “There are 650 district hospitals, 3,000 taluk [subdistrict] hospitals, and more than 23,000 primary health centers in the country. We must aim to connect all these in phases—first the district hospital connected to speciality hospitals in major cities, then the taluk-level hospitals, and finally the primary health centers, so that nobody, irrespective of his location, is deprived of lifesaving specialty consultation.” When the network grows, it may even include private hospitals as well as hospitals in Asia and Africa. Although the network will initially be used for teleconsultation and postoperative consultation, in the future it may accommodate even telesurgery and telerobotics.

## The Impact So Far

Starting with pilot projects in the year 2001, together with a “proof-of-concept” technology demonstration, ISRO has established the facility in nearly 60 remote hospitals, which have been connected with 20 super-specialty city hospitals. [ 13]. A report presented at the Rajya Sabha (the House of States, or Upper House) of the Parliament of India suggested that the initial results of India's telemedicine initiative are encouraging [ 14]. The report states that several telemedicine projects in India have been successfully interlinked—for example, the Andaman and Nicobar Islands telemedicine project links the G. B. Pant Hospital at Port Blair with Shri Ramachandra Medical College and Research Institute, Chennai, while in Karnataka, Narayana Hrudayalaya is connected to District Hospital, Chamarajnagar and Vivekananda Memorial Hospital, Saragur.

Adding to these early reports of successful linkage, there are also reports that telemedicine has helped to save lives in crowded pilgrimage centres and military outposts connected with mobile telemedicine units. For example, the Amrita Telemedicine Programme reports that on 13 January 2003, the programme's first remote telesurgery procedure was performed [ 15]. The Amrita Emergency Care Unit at Pampa was able to save the life of a pilgrim by a telesurgical procedure using the local telemedicine facility. The cardiothoracic surgeon guided the procedure remotely, and the pediatric cardiologist at Pampa performed the procedure. Mobile telemedicine units were also rushed to the coasts and islands of India after the 2004 tsunami to provide medical consultation and relief to the affected people [ 16].

There are other indications that the telemedicine initiative may have had a positive impact. ISRO's annual report for 2004–2005 states: “More than 25,000 patients have so far been provided with teleconsultation and treatment. An impact study conducted on a thousand patients has revealed that there is a significant cost saving in the system since the patients avoid expenses towards travel, stay, and for treatment at the hospitals in the cities” [ 8]. Dr. Devi Shetty, a cardiac surgeon and the Chairman of Narayan Hrudayalya, a hospital that has served thousands through telemedicine, said: “We have treated 17,400 patients using telemedicine connectivity in various parts of India, mainly from rural India, and [a] few patients from outside India. We use both satellite as well as ISDN connectivity. Now, with the Indian Space Research Organisation, which is our associate in this project giving us the satellite connection free of cost, we have a [larger] game plan of offering health care to African and other Asian countries.”

## The Challenges and Controversies

The telemedicine initiative in India has not been free of challenges and controversies. “There are inevitable difficulties associated with the introduction of new systems and technologies,” according to Sathyamurthy. “There are some who needlessly fear that they will lose their jobs. Although the systems are user-friendly, there are others who are affected by the fear of the unknown in handling computers and other equipment. There is a feeling that the initial investment is high and hence financially not viable.” In addition, there may be technical hitches, such as low bandwidth and lack of interoperability standards for software.

Discussing HealthSAT, Dr. D. Lavanian, an Indian expert in telemedicine affiliated with the Apollo Telemedicine Networking Foundation, Apollo Hospitals, Hyderabad, India, said: “[HealthSAT] is excellent, but some questions remain. Presently HealthSAT connectivity is expected to be given free of charge to certain government entities. This is unsatisfactory as a large percentage of health care in India is by private entities.” Dr. Lavanian added: “On my requesting to ISRO to open up the same to the private health industry, of course for a fee, I have not received any positive answer. This means that a large percentage of the population of India will be denied healthcare via telemedicine.”

These difficulties can probably be surmounted. In the late 1980s, when computers came to India, similar kinds of problems were seen in different parts of the country. That is, people showed technophobia and expressed their fears that computers would cause unemployment and would also be prohibitively expensive. But the country overcame these challenges and fears, and eventually became a superpower in the field of knowledge and information technology [ 17].

## Conclusion

With the aid of HealthSAT, India's telemedicine initiative has the potential to provide specialized health care to millions of poor Indians. This potential was well summed up by Dr. Devi Shetty: “In terms of disease management, there is [a] 99% possibility that the person who is unwell does not require [an] operation. If you don't operate you don't need to touch the patient. And if you don't need to touch the patient, you don't need to be there. You can be anywhere, since the decision on healthcare management is based on history and interpretation of images and chemistry … so technically speaking, 99% of health-care problems can be managed by the doctors staying at a remote place—linked by telemedicine.”

## Abbreviation

ISRO: Indian Space Research Organization
